# First Draft Genome Sequence of *Salmonella enterica* Serovar Gallinarum Strain VTCCBAA614, Isolated from Chicken in India

**DOI:** 10.1128/genomeA.01221-15

**Published:** 2015-10-22

**Authors:** R. K. Vaid, N. Jindal, T. Anand, B. C. Bera, T. Riyesh, N. Virmani, S. Barua, Renu Gupta, N. K. Mahajan, C. G. Joshi, R. K. Singh

**Affiliations:** a Veterinary Type Culture Collection, ICAR-National Research Centre on Equines, Hisar, Haryana, India; b Department of Veterinary Public Health and Epidemiology, College of Veterinary Sciences, Lala Lajpat Rai University of Veterinary and Animal Sciences, Hisar, Haryana, India; c ICAR-National Research Centre on Equines, Hisar, Haryana, India; d Department of Animal Biotechnology, College of Veterinary Science and Animal Husbandry, Anand Agricultural University, Anand, Gujarat, India

## Abstract

*Salmonella enterica* subsp. *enterica* serovar Gallinarum biovar Gallinarum causes fowl typhoid (FT), which results in huge economic losses to poultry farmers in India. We report the draft genome sequence of *Salmonella* biovar Gallinarum strain VTCCBAA614, isolated from a chicken in an FT affected broiler flock.

## GENOME ANNOUNCEMENT

Fowl typhoid (FT) is an acute septicemic disease of pullets and adult poultry caused by a nonmotile invasive avian pathogen, *Salmonella enterica* subsp. *enterica* serovar Gallinarum biovar Gallinarum (1). The disease is responsible for considerable economic losses in breeding and commercial laying flocks causing high morbidity and acute mortality in Asia, although disease incidence may be underreported elsewhere (2–4). Disease transmission occurs horizontally through the spread of infectious agents from carrier birds via contaminated feed and water, although transovarian transmission also takes place (5). The pathological changes in acute FT are primarily observed in the liver (1).

The serogroup D nonmotile *Salmonella* biovar Gallinarum is a chicken-adapted biotype capable of causing systemic infection rather than enteritis in birds, in contrast to its motile ancestor *S. enteritidis* (6). The analysis of the close phylogenomic relationship between *Salmonella* biovar Gallinarum and *S. enteritidis* has elucidated a common ancestry (7); however, strains with intermediate biochemical markers among nonmotile salmonellae have been observed (8). The disease may have been eradicated in North America and Europe, but it is endemic throughout India (2, 3, 9). This is the first whole-genome sequence of an FT outbreak *Salmonella* biovar Gallinarum isolate from India. Strain VTCCBAA614 was isolated from the liver of a broiler chicken (*Gallus gallus domesticus*) (2).

Sequencing was achieved by 454 pyrosequencing of a shotgun library and assembled *de novo* using Newbler version 2.6. A total of 2,23,420 reads of 427 bp were generated using the GS FLX Titanium system, giving ~20× coverage. The data generated 92 contigs with an average contig size of 61,644 bp and a largest contig size of 3,02,540 bp. The total size of the genome was 46,86,634 bp, with an *N*_50_ of 1,25,446 bp and a Q40 of 99.75%. The annotation was carried out against strain Ty2 using the RAST server, which showed GC content of 52.20% with 4,581 predicted genes (10) The PGAP analysis data contains 4,186 coding sequences (CDSs), 314 pseudogenes, 4 rRNAs, 66 tRNAs, 11 ncRNAs, and 204 frameshifted genes. The Pathosystems Resource Integration Center (PATRIC) annotation reported 173 pseudogenes and 675 hypothetical proteins (11).

SEED subsystem analysis revealed genes involved in multifarious roles, including virulence, adhesion, bacteriocins, and resistance to antimicrobials and toxic compounds (12). Antimicrobial resistance in *Salmonella* biovar Gallinarum is an emerging concern (13). Strain VTCCBAA614 revealed 55 genes for antimicrobial resistance, including a lactam utilization protein, penicillin-binding protein, polymyxin resistance protein, macrolide-specific efflux protein, and proteins responsible for flouroquinolone and tetracycline resistance.

Six other genomes of *Salmonella* serovar Gallinarum strains SG9, 287/92, FCAV198, CDC1983-67, 9184, and RKS5078 have been sequenced from North America, Brazil, and the United Kingdom, and VTCCBAA614 is the only strain sequenced from the Asian region. Although the genomic relatedness of *Salmonella* serovar Gallinarum strains from distinct geographies is open to comparison, a greater number of sequences will bring further clarity to the pseudogene contents of the two nonmotile strains. The genomic data may be useful in providing insight into the pathogenesis, biology, and mechanisms of host tropism, and may provide clues for smart vaccines.

### Nucleotide sequence accession numbers.

This whole-genome shotgun project has been deposited at GenBank under the accession number JSWQ00000000. The version described in this paper is the first version, JSWQ01000000.
